# Epigenetic regulation of CD133 and tumorigenicity of CD133 positive and negative endometrial cancer cells

**DOI:** 10.1186/1477-7827-8-147

**Published:** 2010-12-01

**Authors:** Anne M Friel, Ling Zhang, Michael D Curley, Vanessa A Therrien, Petra A Sergent, Sarah E Belden, Darrell R Borger, Gayatry Mohapatra, Lawrence R Zukerberg, Rosemary Foster, Bo R Rueda

**Affiliations:** 1 Vincent Center for Reproductive Biology, Vincent Department of Obstetrics and Gynecology, Massachusetts General Hospital, Boston, MA 02114, USA; 2 Department of Obstetrics, Gynecology and Reproductive Biology, Harvard Medical School, Boston, MA 02115, USA; 3 Division of Hematology-Oncology, Massachusetts General Hospital Cancer Center, Boston, Massachusetts 02114, USA; 4 Department of Molecular Pathology, Massachusetts General Hospital, Boston, MA 02114, USA; 5 Department of Pathology, Harvard Medical School, Boston, MA 02115, USA; 6 Gynecologic Oncology Division, Vincent Department of Obstetrics and Gynecology, Massachusetts General Hospital, Boston, MA 02114, USA

## Abstract

**Background:**

Recent data provide significant evidence to support the hypothesis that there are sub-populations of cells within solid tumors that have an increased tumor initiating potential relative to the total tumor population. CD133, a cell surface marker expressed on primitive cells of neural, hematopoietic, endothelial and epithelial lineages has been identified as a marker for tumor initiating cells in solid tumors of the brain, colon, pancreas, ovary and endometrium. Our objectives were to assess the relative level of CD133 expressing cells in primary human endometrial tumors, confirm their tumorigenic potential, and determine whether CD133 expression was epigenetically modified.

**Methods:**

We assessed CD133 expression in primary human endometrial tumors by flow cytometry and analyzed the relative tumorigenicity of CD133+ and CD133- cells in an *in vivo *NOD/SCID mouse model. We assessed potential changes in CD133 expression over the course of serial transplantation by immunofluorescence and flow cytometry. We further examined CD133 promoter methylation and expression in normal endometrium and malignant tumors.

**Results:**

As determined by flow cytometric analysis, the percentage of CD133+ cells in primary human endometrial cancer samples ranged from 5.7% to 27.4%. In addition, we confirmed the tumor initiating potential of CD133+ and CD133^- ^cell fractions in NOD/SCID mice. Interestingly, the percentage of CD133+ cells in human endometrial tumor xenografts, as evidenced by immunofluorescence, increased with serial transplantation although this trend was not consistently detected by flow cytometry. We also determined that the relative levels of CD133 increased in endometrial cancer cell lines following treatment with 5-aza-2'-deoxycytidine suggesting a role for methylation in the regulation of CD133. To support this finding, we demonstrated that regions of the CD133 promoter were hypomethylated in malignant endometrial tissue relative to benign control endometrial tissue. Lastly, we determined that methylation of the CD133 promoter decreases over serial transplantation of an endometrial tumor xenograft.

**Conclusions:**

These findings support the hypotheses that CD133 expression in endometrial cancer may be epigenetically regulated and that cell fractions enriched for CD133+ cells may well contribute to endometrial cancer tumorigenicity, pathology and recurrence.

## Background

Endometrial cancer is the most common cancer of the female reproductive organs in the United States [1-5]. The continual remodeling of the endometrial lining at menses strongly argues for the presence of a stem/progenitor cell population with regenerative capabilities. This is further supported by studies of the benign endometrium in primate models and clonogenicity assays of human derived uterine cells [6-10]. Mouse studies utilizing pulse-chase experiments to demonstrate evidence of label retaining cells in the uterus [11,12] provide additional functional evidence to support this concept. Thus, it has been proposed that an aberrant stem/progenitor cell or a cell that regains some stem-like properties can contribute to pre-malignant endometrial hyperplasia and/or endometrial cancer [6,7,13].

Several investigators have identified putative stem/progenitor cells in solid tumors and cancer cell lines within the side population (SP), which is distinguished by differential efflux of Hoechst 33342 dye via verapamil-sensitive multidrug resistance transporters [14-16]. Our previous work [13] identified a tumorigenic SP within a human endometrial cancer cell line that displayed increased chemoresistance and quiescence *in vitro *relative to its non-SP counterpart. Hubbard and colleagues [7] demonstrated endometrial cancer cells with clonogenic, self-renewing, differentiating and tumorigenic properties further supporting the hypothesis that a cancer stem cell population may be responsible for seeding tumors or metastatic lesions.

Tumor initiating cells have been identified in leukemia [17] and in a variety of solid tumors [18-23] based on differential expression of one or more cell surface markers, suggesting that tumor initiating cell heterogeneity exists for each specific tumor type. The CD133 (human Prominin-1, AC133) cell surface antigen was originally identified in hematopoetic stem cells [24,25] and shown to be expressed on primitive cells of neural, endothelial and epithelial lineages. Several investigators have identified CD133 as a potential tumor initiating cell marker in solid tumors of the brain [18], prostate [19], colon [20] and more recently the ovary [21,22] and endometrium [23]. CD133^+ ^cells have been associated with an increase in *in vivo *tumor initiation [18,26], asymmetric cell division and increased resistance to chemotherapeutic drugs [26], as compared to CD133^- ^cells. Additionally, SP fractions have been reported to be enriched for CD133^+ ^cells [27]. In the ovary, CD133^+ ^cells have been associated with the presence of primary disease rather than with normal ovaries or metastatic omental lesions [22] and sorted ovarian CD133^+ ^tumors cells form more aggressive tumor xenografts compared to their CD133^- ^progeny [26]. Similarly, CD133^+ ^cells isolated from endometrioid adenocarcinomas were resistant to cisplatin- and paclitaxel-induced cytotoxicity [23]. When serially transplanted into NOD/SCID mice, CD133^+ ^cells were capable of initiating tumor formation that resembled the phenotype of the original tumor [23]. Together these data support the hypothesis that CD133 is expressed by human endometrial cancers and may serve as a marker of more tumorigenic cells.

Recent studies have indicated that CD133 expression and antigenic potential [28] may be regulated in part by histone modification [26], DNA methylation [29,30] and/or glycosylation [28,31]. Regulation of CD133 expression by DNA methylation-dependent mechanisms has been observed in glioblastoma [24,32], colorectal cancer [29,30] and ovarian cancer [26]. Our objectives were to confirm the tumorigenic potential of CD133^+ ^cells in immunocompromised mice, assess whether CD133 levels increased in serially transplanted tumors concurrently with their accelerated tumor formation rate and determine whether methylation status was associated with changes in the levels of CD133.

Our results confirm that endometrial tumors contain CD133^+ ^cells, which can generate new tumors following injection in NOD/SCID mice. Cell fractions enriched for CD133^+ ^cells gave rise to tumors at a faster rate than CD133^- ^cell populations at fewer cell numbers injected. Interestingly, the level of CD133^+ ^cells, as determined by immunofluorescence, in tumor explants appeared to be enriched with sequential serial transplantation of the tumor cells although this apparent increase in CD133 levels was not consistently detected by flow cytometry. Despite the fact that the percentage of CD133^+ ^cells varies widely in established endometrial cancer cell lines, the level of mRNA encoding CD133 was elevated following treatment with the demethylating agent, 5-aza-2'-deoxycitidine (5-aza-dc), suggesting that methylation status may be important in the regulation of CD133 expression or epitope presentation. This concept was further supported by evidence that the CD133 promoter is hypomethylated in primary endometrial cancer tissue compared to benign endometrium. Collectively, these data support the hypothesis that CD133 may serve as a marker to assess potential tumorigenicity of endometrial cancer cells and that its expression levels are controlled in part through epigenetic regulation.

## Methods

### Human primary endometrial epithelial cell isolation

All primary human uterine tissues were collected in accordance with the policies of the Massachusetts General Hospital (MGH) Institutional Review Board. A subset of samples was collected utilizing the MGH GYN tissue repository after obtaining informed consent and a second subset was collected as anonymized discarded tissue. As per IRB protocol, the samples (n = 12) were not linked to clinical information. The histological subtype and grade of each sample was retrospectively assessed by an MGH pathologist (LRZ). The details of the tumor samples used in this study are listed in Table 1.

**Table 1:** Pathological evaluation of the human endometrial tumor samples used

Sample ID	Pathology
T1	Grade 1 Endometrioid
T2	Grade 3 Endometrioid
T3	Grade 3 Endometrioid
T4	Grade 1 Endometrioid
T5	Grade 1 Endometrioid
T6	Grade 1 Endometrioid
T7	Grade 2-3 Endometrioid
T8	Grade 3 Endometrioid
T9	Grade 1 Endometrioid

Endometrial carcinoma tissues were minced to yield 2 mm^3 ^pieces and incubated with agitation in HBSS (Cambrex Corp., East Rutherford, NJ)/2% FBS (HyClone, Logan, UT)/1 mM EDTA (Sigma-Aldrich, St. Louis, MO) containing 1 mg/ml collagenase Type II (Sigma) and 0.025% DNase I (Sigma) at 37°C for 1 hour. Following this incubation, the supernatant was removed and discarded. The remaining digested tissue was washed with Dulbecco's phosphate buffered saline (PBS) (Cambrex) and resuspended in DMEM medium (Mediatech Inc., Herndon, VA) containing 2% FBS, added L-glutamine (100 U/ml), penicillin (1%), streptomycin (1%) and 2.5 μg/ml amphotericin B (Sigma), and incubated in a T75 flask at 37°C, 5% CO_2 _in a humidified chamber for 1 hour. The flask was rotated at 20-minute intervals during this incubation period to maximize binding of stromal cells to the vessel walls. Endometrial epithelial cells, which make up the bulk of the cells in the non-adherent cell population, were then removed from the flask. The number of non-viable cells, as determined by Trypan blue staining (Mediatech Inc.), was assessed and these cells were eliminated from the suspension using the Dead Cell Removal Kit (Miltenyi Biotec Inc., Auburn, CA) as necessary.

### *In vivo *endometrial tumorigenesis assay

All experiments utilizing mouse models were reviewed and approved by the MGH Institutional Animal Care and Use Committee and were performed in strict accordance with the NIH Guide for the Care and Use of Laboratory Animals. Six to twelve week old female NOD/SCID mice (strain NOD.CB17-Prkdcscid/J, Jackson Laboratory, Bar Harbor, ME) were used for all injections of human primary endometrial tumor epithelial cells. Defined numbers of isolated primary endometrial epithelial cells were suspended in 1:1 PBS/Matrigel^® ^(BD Biosciences, San Jose, CA) and subcutaneously (s.c.) injected into the right dorsal side of NOD/SCID mice. Control animals were simultaneously injected with 1:1 PBS/Matrigel^® ^only. Tumor development was assessed bi-weekly.

### Tumor initiating capacity of transplanted endometrial cells

#### Xenograft processing

Mice bearing tumors generated following injection of primary endometrial tumor epithelial cells were euthanized by CO_2 _inhalation. The generated tumors were isolated aseptically, minced to yield 2 mm^3 ^pieces and incubated with agitation at 37°C for 30 minutes in HBSS/2% FBS, 1 mM EDTA containing 1 mg/ml collagenase Type II, 0.025% DNase I and 2.5 μg/ml amphotericin B. Cells were filtered through a 100 μm mesh filter (BD Biosciences) and washed 3 × 5 minutes in HBSS/2% FBS/1 mM EDTA. Pelleted cells were resuspended in ACK lysis buffer (Cambrex) for 1 minute at room temperature to lyse red blood cells. The remaining cells were washed in HBSS, resuspended in HBSS/2% FBS/1 mM EDTA and layered over Ficoll-Paque™ PLUS (GE Healthcare Bio-Sciences Corp., Piscataway, NJ). Non-viable cells were eliminated from the suspension using a Dead Cell Removal Kit (Miltenyi Biotec Inc.). H-2K^d+ ^mouse cells were removed using a FITC conjugated antibody (BD Biosciences) and MACS^® ^LD separation columns (Miltenyi Biotec Inc) as per manufacturers' recommendations. H-2K^d- ^cells were suspended in 1:1 PBS/Matrigel^® ^and injected s.c. into the right dorsal side of NOD/SCID mice. Tumor development was assessed bi-weekly.

#### CD133 isolation via magnetic beads

Isolated H-2K^d- ^cells were separated into CD133^+ ^and CD133^- ^fractions using CD133 microbeads (Miltenyi Biotec Inc.) and MACS^® ^LD separation columns as per manufacturers' recommendations. To acquire a pure CD133^- ^population, CD133^- ^cells were passed twice through separate LD columns. Defined numbers of CD133^+ ^and CD133^- ^cells were injected into NOD/SCID mice as previously described.

### Flow cytometry

#### CD133 profiling

To examine expression of CD133, single human endometrial cells from primary or transplanted tumors were isolated as outlined previously [33]. Following incubation with FcR blocking reagent (Miltenyi Biotec Inc.) to reduce unwanted binding of antibody to Fc receptor-expressing cells, tumor cells were resuspended in PBS/2% FBS/1 mM EDTA and stained with 0.5 μg anti-CD133 (phycoerythrin (PE)-conjugated; Miltenyi Biotec Inc.). Respective IgG isotype antibodies were included as negative controls. Non-viable cells were excluded using the LIVE/DEAD Fixable Dead Cell Stain kit (Invitrogen, Carlsbad, CA) as per manufacturer's recommendations. For primary tumors, CD31^+ ^and CD45^+ ^cells were excluded using FITC-conjugated CD31 and CD45 antibodies (Miltenyi Biotec Inc.) Similarly, for xenograft tumors, H-2K^d+ ^cells were eliminated using a FITC-conjugated H-2K^d ^antibody. After washing in PBS/2% FBS/1 mM EDTA, cells were fixed by incubation in 4% paraformaldehyde for 60 minutes and analyzed using a LSRII (BD Biosciences) within 24 hours. Data were analyzed using FlowJo version 8.2 software.

#### Cell sorting

Single cell suspensions derived from endometrial xenograft tumors were stained with anti-CD133 as described. CD133^+ ^and CD133^- ^cell populations were separated using a FACSAria flow cytometer, with post-sort analysis performed to confirm population purity. Sorted cell populations were serially diluted in 1:1 PBS:Matrigel^® ^and injected s.c. into female NOD/SCID mice.

### Oligonucleotide array CGH (aCGH)

Array CGH was performed to determine if there were any DNA copy number changes in cells derived from serially transplanted endometrial tumors and from tumors generated from CD133^+ ^and CD133^- ^injected cells using Agilent Human 105K oligonucleotide microarrays as per manufacturer's instructions http://www.home.agilent.com/agilent/home.jspx. Genomic coordinates for this array are based on the NCBI build 36, March 2006 freeze of the assembled human genome (UCSC hg18), available through the UCSC Genome Browser. This array provides an average spatial resolution of 21.7 kb.

Genomic DNA was isolated from primary tumors using standard protocols. For array hybridizations, 5 micrograms each of tumor and normal DNA were digested with Dpn II for 3 hours at 37°C and purified with QIAquick PCR purification columns. One microgram each of purified tumor and normal DNA was labeled with Cy3-dCTP and Cy5-dCTP, respectively, using Bioprime labeling kit (Invitrogen) in accordance with the manufacturer's instructions. Unincorporated nucleotides were removed using Sephadex G-50 columns. Labeled tumor and reference samples were precipitated with 50 micrograms of human Cot-1 DNA and resuspended in 250 microliters of hybridization buffer provided in the Agilent oligonucleotide array CGH kit. Prior to hybridization, probe mixtures were denatured for 5 minutes at 95°C and incubated at 37°C for 30 minutes. Samples were then hybridized onto the oligonucleotide array in the Agilent SureHyb microarray hybridization chamber and hybridization was performed for 42 hours at 65°C. The arrays were disassembled and washed as recommended by the manufacturer. Slides were dried and scanned with an Axon 4000B microarray scanner using GenePix Pro 4.0. Microarray images were analyzed and data points generated using the Feature Extraction software (version 9.1, Agilent Technologies) with linear normalization (protocol-v4_91). Data were subsequently imported into CGH Analytics software (version 3.4.40, Agilent Technologies). Detection of gains and losses were based on the z-score algorithm (threshold 2.5) and visual inspection of the log2 ratios. Log2 ratios ≥0.4 in at least five consecutive probes were considered a reliable copy number alteration. Probes with log2 ratios greater than 2 were considered highly amplified.

### CD133 immunofluorescence

Immunofluorescence was carried out on 6 micron sections of formalin-fixed, paraffin embedded (FFPE) biopsies from primary human endometrial and xenograft tumors. Antigen retrieval was carried out using 10 mM Citrate (pH 6.0). After blocking, sections were incubated with CD133 antibody (K-18; Santa Cruz) or Goat Negative Control at 1:50 dilution overnight at 4°C. Donkey anti-goat Alexa Fluor 568 was used as secondary antibody. DAPI was used to stain nuclei.

### Methylation analysis of CD133 promoter

Human endometrial cancer cell lines were treated with either vehicle or 5-aza-2'-deoxycytidine for 72 hours. CD133 expression was evaluated by RT-PCR and flow cytometry.

### Expression of CD133 mRNA in benign and malignant samples

FFPE tissue specimens were obtained from the MGH Pathology archives with IRB approval. Histological review was performed by a pathologist on hematoxylin and eosin stained slides to confirm pathology and designate areas of tumor on relevant slides. Tissue was macrodissected from serial 5 micron slides and total nucleic acids were extracted using a custom fully-automated platform based on the FormaPure System (Beckman Coulter Genomics, Danvers, MA) and Beckman Coulter Biomek NX^P ^workstation.

### RT-PCR analysis

Single stranded cDNA was prepared with the Superscript First-Strand System (Invitrogen). Mock reactions were prepared under the same conditions but lacked reverse transcriptase. The cDNAs were amplified by PCR using the following primer sets: CD133-3: 5'-AGCTTCTCTGGATTTTGCTCA-3' (forward) and 5'-CACAGAAAGACATCAACAGCAG-3' (reverse); CD133-5: 5'-CAGAAGGCATATGAATCCAAAA-3' (forward) and 5'-CTGTCGCTGGTGCATTTCT-3' (reverse); β-actin: 5'-CTTCCAGCCTTCCTTCCTG-3' (forward) and 5'-TTGGCGTACAGGTCTTTGC-3' (reverse). The PCR products were analyzed by 1.5% agarose gel elctrophoresis.

### Bisulfite-treated genomic DNA sequencing

Genomic DNA was isolated from normal endometrium and endometrial tumor using the high pure PCR template preparation kit (Roche). Bisulfite treatment was carried out using EZ DNA methylation kit (Zymo Research). Mapping of methylated cytosines was carried out by bisulfite-treated genomic DNA sequencing. The CD133 promoter region that encompasses the CpG island was divided into three regions defined by the PCR primers used to amplify the bisulfite-treated DNA. Ten individual clones were analyzed per region and tissue sample. The percentage of CpG methylation within each region was compared between benign and malignant tissue. All malignant tissues analyzed (n = 3, T7-T9) were endometrioid endometrial adenocarcinoma. Benign endometrial tissue (n = 3) was derived from pre-menopausal women with no evidence of malignant disease who were undergoing hysterectomy as a result of extensive uterine fibroids.

### Statistical analysis

Student's t test was used for statistical comparisons where appropriate. A p value of < 0.05 for the t test was considered to be statistically significant.

## Results

### Assessment of CD133 expression in primary human endometrial tumors

**Figure 1:**
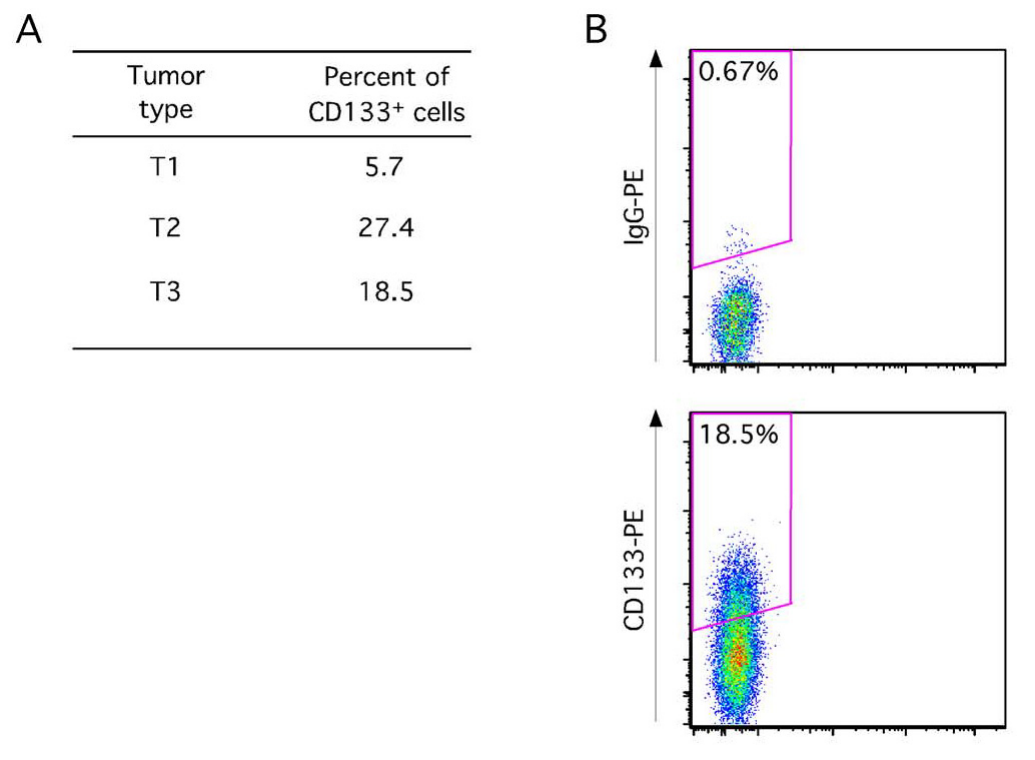
**CD133 is expressed in primary human endometrial tumors**. Panel A shows the percentage of CD133^+ ^cells in three independent primary human endometrial tumors (T1-T3) as determined by flow cytometry. Panel B is a representative flow cytometric analysis of the T3 primary endometrioid tumor. CD133^+ ^and matched IgG isotype stained populations are shown in panel B.

Other investigators have reported the presence of CD133 expressing cells in gynecologic tumors [21-23,26,33,34]. We analyzed CD133 expression in primary endometrial tumors dissociated to single cell suspensions that were depleted of CD31^+ ^endothelial cells and CD45^+ ^hematopoietic cells. The percentage of CD133^+ ^cells within the total tumor cell population was determined by flow cytometric analysis using a phycoerythrin (PE)-conjugated anti-CD133 antibody. The results from analyses of three independent primary endometrial tumors (T1-T3) are shown in Figure 1A. Figure 1B is a representative example of the flow data generated in these analyses. CD133^+ ^cells comprised 5.7%-27.4% of the total tumor cell population in the analyzed primary tumors, which is similar to the range reported by Rutella and colleagues [23].

### Serial transplantation of human endometrial tumor xenografts

**Figure 2:**
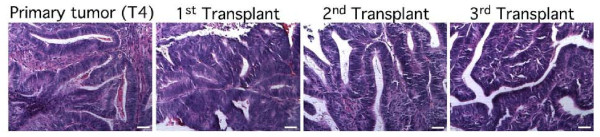
**The original endometrial tumor histopathology is maintained across the serial transplantation process**. Hematoxylin and eosin stained sections from a primary human endometrial tumor (T4) and its xenograft tumors propagated over several rounds of serial transplantation in NOD/SCID mice are shown. The histology of the parent endometrioid endometrial tumor is maintained by the tumors generated at each serial passage. Scale bar, 50 μm.

We have developed an *in vivo *experimental system in which primary human endometrial tumors are propagated and expanded through serial passaging in immunocompromised NOD/SCID mice. We have used this system in our studies of tumor initiating cells in ovarian cancer [33] and have successfully adapted it for analysis of similar populations in human endometrial tumors. The system allows us to expand the limited amount of primary endometrial tumor tissue sample obtained at the time of surgery in the absence of *in vitro *culturing and thereby avoids potential artifactual influences of culture or medium conditions. To date, we have carried out 3-4 rounds of serial transplantation with seven independent primary human endometrial tumors of varying grade and histological subtype. In all cases, tumor histopathology has been maintained across the serial passaging process (Figure 2), indicating that xenograft tumors expanded in this *in vivo *system reliably model the original primary tumor.

### Differential tumor initiating capacity of CD133^+ ^cells

We used our serial transplantation system to determine if CD133 identifies tumor initiating cells in human endometrial cancer as previously reported [33]. In pilot experiments, we utilized a magnetic bead based separation method to generate tumor-derived cell populations enriched for CD133^+ ^or CD133^- ^cells, which were then injected into NOD/SCID mice to assess the relative tumorigenicity of each fraction. Following injection of the isolated cell fractions, tumor developed in 3/3 mice injected with 50,000 CD133^+ ^cells with a latency of 43 days. In contrast, only 1/3 mice injected with CD133^- ^cells developed a tumor and the latency was 89 days.

Our results from the magnetic bead isolation studies suggested that CD133^+ ^cells derived from human endometrial tumors had enhanced tumor initiation capacity relative to their CD133^- ^counterparts. To further investigate this possibility, we carried out cell sorting experiments to generate highly purified populations of CD133^+ ^and CD133^- ^cells and analyzed the relative tumorigenicity of each population as well as that of the unsorted parent tumor cell population following injection in NOD/SCID mice. The results of one such experiment are shown in Table 2. In this study, injection of as few as 500 CD133^+ ^cells resulted in tumor formation. In contrast, injection of 20-200 times more bulk unsorted or CD133^- ^cells were required to generate tumors with similar latency. These findings support previous studies [23] showing that CD133^+ ^cells derived from human endometrial tumors have an increased capacity to form tumors.

**Table 2:** Tumorigenic capacity of CD133+ and CD133- endometrial transplanted tumor cells in vivo

	**Cell dose**^**a**^**, tumor formation**^**b **^**and latency (days)**^**c**^				
	
T4	**1 × 10**^**5**^	**1 × 10**^**4**^	**1 × 10**^**3**^	**5 × 10**^**2**^	**1 × 10**^**2**^
CD133^+^	1/1 (54)	1/1 (56)	1/1 (61)	1/1 (61)	0/1
CD133^-^	1/1 (89)	0/1	0/1	0/1	0/1
Bulk	1/1 (61)	1/1 (61)	0/1	0/1	0/1

^a^Number of cells injected

^b^Number of tumors formed from number of cells injected

^c^Days from initial injection to palpable tumor formation

### Assessment of CD133 expression in serially transplanted human endometrial tumors

**Figure 3:**
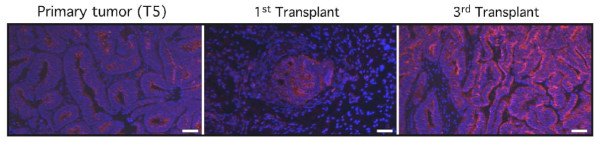
**CD133 expression increases over the course of serial transplantation**. Relative CD133 expression was assessed in a human primary endometrioid endometrial tumor (T5) and its serially passaged xenografts by immunofluorescence. The protein was predominantly detected on the surface of endometrial cells and its expression increased with serial transplantation Scale bar, 50 μm.

We previously observed that the time to tumor formation in our serial transplantation system decreased with each successive transplant despite fewer cells being injected (data not shown). This was seen with all primary tumors that were successfully propagated *in vivo*. One explanation of this phenomenon is that serial transplantation enriches for a tumor initiating cell population. To determine if the frequency of CD133^+ ^cells increases over the course of serial transplantation, we first assessed relative CD133 expression in a primary tumor and first and third transplantation xenografts of the same tumor by immunofluorescence. As shown in Figure 3, CD133 expression was retained in the transplanted tumors. More interestingly, we observed a qualitative increase in the relative proportion of CD133^+ ^cells with successive transplantation although this increase was not consistently evident in flow cytometric analyses of CD133 expression in serial transplants (data not shown).

### aCGH analysis of serially transplanted tumors

**Figure 4:**
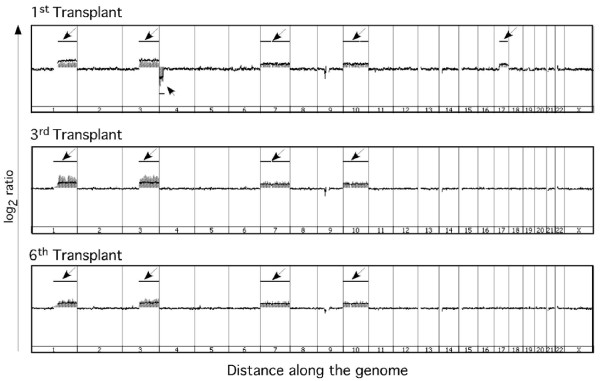
**Serial transplantation does not generate significant genomic alterations in endometrial tumors**. The integrity of the genome was assessed in the first, third and sixth serially transplanted xenograft tumors derived from endometrioid endometrial tumor sample T6 by array comparative genomic hybridization (aCGH). As shown, the genomic profiles remain virtually unchanged across the transplant process. Arrows indicate regions of increased and decreased DNA copy numbers detected on chromosomes 1-22 and the X chromosome.

The observed qualitative increase in the relative proportion of CD133^+ ^cells in successive serial transplants may result from genomic alterations that arise over the transplantation process. To investigate this possibility, we carried out array comparative genomic hybridization (aCGH) analyses of xenograft tumors generated by serial transplantation of an endometrioid endometrial tumor. There was no evidence of gene amplification in chromosome region 4p15.32 which encompasses the CD133 locus (Figure 4). Interestingly, we observed no other major changes in copy number over serial passaging suggesting considerable genomic stability over the transplantation process.

### CD133 expression is regulated by methylation

**Figure 5:**
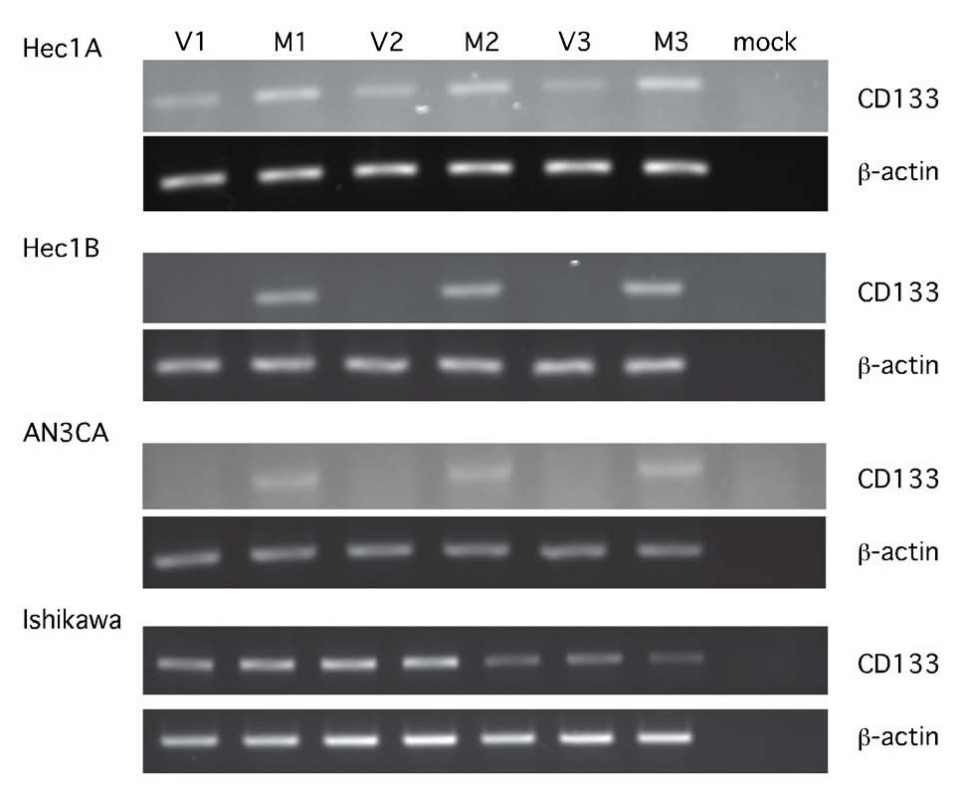
**CD133 expression is regulated by methylation in human endometrial cancer cell lines**. CD133 expression was assessed in triplicate by RT-PCR in the Hec1A, Hec1B, AN3CA and Ishikawa cell lines following treatment *in vitro *with either vehicle (0.005% DMSO, V1-V3) or 5 μM 5-aza-deoxycytidine (M1-M3). CD133 mRNA levels were increased in Hec1A, Hec1B and AN3CA cells following treatment with 5-aza-deoxycytidine. β-actin expression was used to confirm equivalent RNA concentrations in all samples.

Previous reports have suggested that CD133 expression in other tumor types [26] may be epigenetically regulated. To determine if CD133 is regulated by methylation in endometrial cancer, we first analyzed CD133 expression in four different human endometrial cancer cell lines. Cells were treated with either 5 μM 5-aza-dc or vehicle for 72 hours. Following treatment with 5-aza-dc, CD133 mRNA levels were increased relative to the vehicle controls in three of the four tested cell lines (Figure 5), suggesting that CD133 expression could, at least in part, be regulated by methylation. This was further supported by the flow cytometric analysis of CD133 protein levels (Table 3) following treatment with either vehicle or 5-aza-dc in which we observed a modest increase in CD133 protein expression in three of the cell lines.

**Table 3:** Flow cytometric analysis of CD133 expression levels after treatment with 5-aza-dC

Cell Lines	Vehicle	5-aza-dC
HEC1A	0.8%	1.5%
HEC1B	0%	10%
An3Ca	0%	0%
Ishikawa	82.6%	87.3%

**Figure 6:**
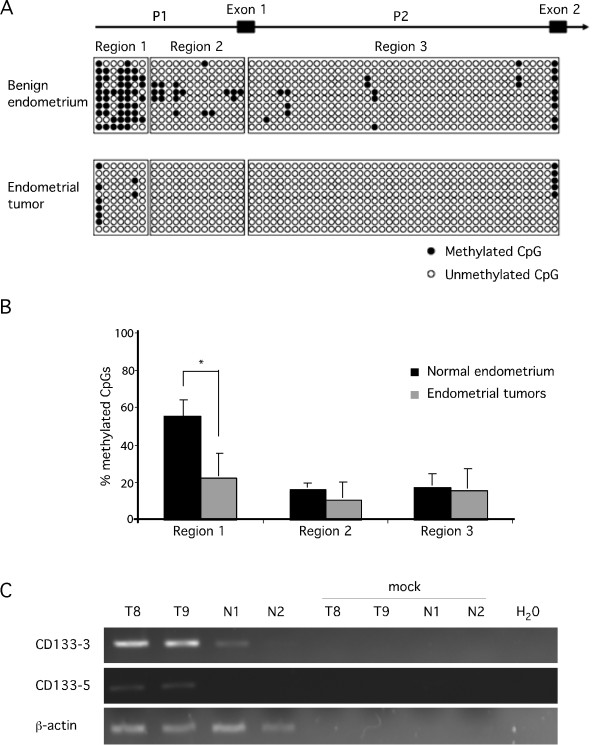
**The CD133 promoter is hypomethylated in malignant endometrial tumors**. Panel A is a schematic depicting the methylation status of the Regions 1-3 of the CD133 promoter in normal endometrium (n = 3) and malignant tumors (samples T7-T9). Ten DNA clones were analyzed from each sample and individual CpG sites are represented by circles. The methylation of any specific CpG sites is indicated by a filled circle. Panel B is a histogram illustrating the relative percentage of methylated CpG sites detected in the analysis shown in panel A. The percentage of methylated CpG sites in Region 1 of the CD133 promoter was significantly decreased (p < 0.01) in malignant endometrial tumors compared to benign endometrium. CD133 mRNA levels in a subset of the same tumor (samples T8 and T9) and normal endometrium (N1 and N2) samples were analyzed by RT-PCR (panel C). Expression of CD133 mRNA was increased in the tumor samples relative to the benign endometrium, consistent with the methylation patterns depicted in panel A. β-actin expression was used to confirm equivalent RNA concentrations in all samples.

**Figure 7:**
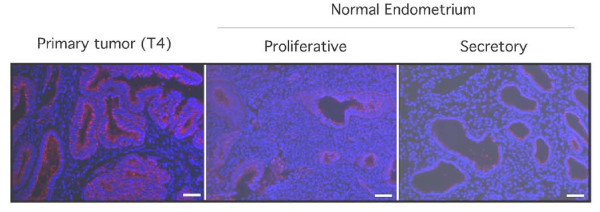
**CD133 protein expression is elevated in endometrial tumors**. The relative expression of CD133 was evaluated in a primary endometrial tumor and proliferative and secretory endometrium by immunofluorescence. There was robust expression of CD133 in a low grade endometrial tumor (T4) compared to normal benign proliferative and secretory endometrium. Scale bar, 50 μm.

To extend these findings, we assessed whether there was evidence of differential methylation of CpG sites in the CD133 promoter in three sets of benign and malignant tissue samples derived from women who either had no evidence of endometrial cancer or had been diagnosed with endometrial cancer. We focused our analyses on three different regions of the CD133 promoter which had previously been shown to be important for the regulation of CD133 expression [26]. Figure 6A shows the methylation pattern of three regions along the promoter analyzed from 10 individual clones derived from either normal benign endometrium or malignant endometrial tumor. We detected a significant (p < 0.01) decrease in the methylation of promoter region 1 in malignant tumor compared to normal benign endometrium (Figure 6B). This approximately 3-fold drop in CD133 promoter CpG methylation was consistently observed in malignant endometrial tumor samples as compared to endometrium collected from women with no evidence of endometrial cancer. We next analyzed the level of CD133 mRNA in these same benign and malignant samples (Figure 6C) and detected very little if any expression of CD133 mRNA in the benign samples relative to the malignant samples. Finally, we analyzed CD133 expression in benign proliferative and secretory endometrium and in low grade malignant endometrial tissue by immunofluorescence (Figure 7). Although we detected minimal CD133 protein expression in the benign samples, we observed more robust CD133 expression in the malignant tissue, consistent with our methylation analyses.

### CD133 promoter methylation decreases with successive serial transplantation

**Figure 8:**
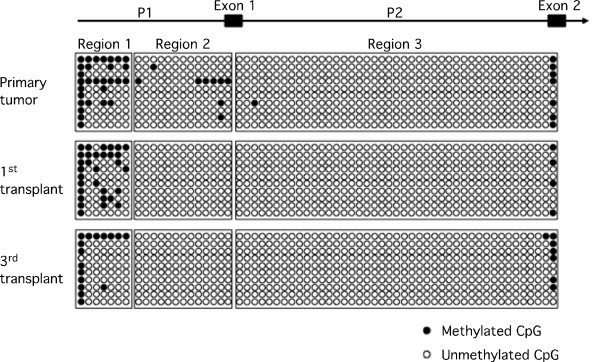
**The CD133 promoter is hypomethylated over serial transplantation**. The methylation status of Regions 1-3 of the CD133 promoter in human primary tumor sample T5 and its corresponding first and third serially transplanted xenografts is depicted. Ten DNA clones were analyzed from each sample and individual CpG sites are represented by circles. The methylation of any specific CpG sites is indicated by a filled circle.

Previous work utilizing serial transplantation models demonstrated that the number of cells required to generate tumor significantly decreased with each subsequent serial transplantation with a concomitant decrease in the time to onset of tumor formation [7,33]. We and others have determined that CD133 expression identifies a tumor initiating cell population in endometrial cancer [13,23,35]. Moreover, our present data suggest that CD133 expression is increased over the course of serial transplantation (Figure 3). We therefore analyzed the methylation status of the CD133 promoter in the same primary tumor sample T5 and its first and third transplanted tumor xenografts. We observed a reduction of methylation in region 1 in the serial transplants as compared to the primary tumor sample (Figure 8). This is the same CD133 promoter region that displayed the most significant differences in methylation in the benign and malignant endometrial tissue samples.

## Discussion

To date, the complement of surface markers utilized for the isolation of tumor initiating cells (also referred to as cancer stem or cancer initiating cells) from solid tumors have varied in breast (CD44^+^, CD24^-/low^, EpCAM^+^, Lineage^- ^[36]), brain (CD133^+ ^[18]), pancreas (CD44^+^/CD24^+^/EpCAM^+ ^[37]), colon (CD133^+ ^[20,38]) and prostate (CD44^+^/α_2_β_1_^hi^/CD133^+ ^[19]) cancer. More recently, gynecological cancer initiating cells have been isolated based on either differential expression of a number of surface antigens (CD44^+^, CD117^+ ^or CD133) or differential exclusion of Hoechst dye [13,16,33,34,39,40]. In studies to date, CD133 appears to be one of the most consistent markers of gynecological cancer initiating cells. While the link to tumorigenicity has been met with some criticism [28], differential CD133 expression has proven to be an effective tool in the isolation of sub-populations of ovarian and endometrial cancer cells with increased tumor forming capacity [22,23,33]. Consistent with previous reports, we detected CD133 expressing cell populations in tumors obtained from patients diagnosed with endometrioid endometrial adenocarcinoma. The CD133^+ ^cells had increased tumor forming capacity relative to their CD133^- ^counterparts. Interestingly, we determined that CD133 expression increased in serially transplanted tumor xenografts when assessed by immunofluorescence although this increase was less consistently evident in flow cytometric analyses. The differences in the results obtained from these analyses may reflect technique-related variation in antigen presentation/epitope exposure as described by others [28]. The inconsistencies in the flow cytometric analyses likely result in part from variability in sample processing and antibody preparation. Our aCGH analyses determined that the increase in CD133 expression across the serial transplants did not result from changes in gene copy number. We did, however, detect a relative decrease in the level of methylation at the CD133 chromosomal locus (data not shown). This observation led us to more thoroughly investigate potential epigenetic regulation of CD133 expression.

The CD133 gene was previously reported to have 5 distinct promoter regions (P1-P5) that are activated in a tissue-specific manner [41]. Promoter regions P1-P3 are located within a CpG island and P1 and P2 are inactive when methylated, suggesting epigenetic regulation of these regions. We first assessed the level of CD133 mRNA in the human endometrial cancer cell lines Hec 1A, Hec 1B, AN3CA and Ishikawa and found it to be highly variable. Treatment with a demethylating agent resulted in an increase in the relative expression of CD133 mRNA in three of the four cell lines, suggesting that CD133 expression in these cell lines is regulated in part by methylation. This hypothesis was further supported by flow cytometric analyses of the same cell lines which determined that CD133 expression was increased relative to vehicle treated controls following treatment with the demethylating agent.

We next investigated the methylation status of the P1 and P2 CD133 promoter regions in primary human endometrial tumors. Our analyses indicated that the P1 promoter region was significantly demethylated in endometrial tumors compared to benign endometrium (Figure 6A). We found no obvious differences in P2 suggesting that this promoter region is not utilized in primary human endometrial tumors. This is in contrast to ovarian tumors, for example, where significant differences in P2 promoter methylation have been reported [26].

We hypothesized that the *in vivo *level of CD133 expression should be elevated in malignant tissue relative to the level in benign tissue. Indeed, we detected a higher level of CD133 protein expression in primary human endometrial tumors compared to expression in benign proliferative and secretory endometrium. Moreover, the relative level of CD133 mRNA was much greater in malignant samples when compared to the levels in benign endometrial samples. Additionally, analysis of the relative methylation in 3 different regions of the CD133 promoter revealed significant hypomethylation of one region in malignant endometrial tumor tissue. Interestingly, we analyzed the methylation status of a primary tumor sample and compared it to its corresponding serial transplants and found the level of CD133 promoter methylation relative to that detected in the primary human endometrial tumor appeared to be reduced over the course of serial transplantation. As noted, the number of cells required to generate tumor significantly decreased with subsequent serial transplantation with a concomitant decrease in the time to onset of tumor formation. Our findings suggest that this may result from increased CD133 expression due to progressive promoter hypomethylation. Future studies designed to determine the transcription factors, co-activators or co-repressors that directly or indirectly function through the methylated CD133 promoter regions to regulate CD133 expression may be of importance.

## Conclusions

Although our results support the hypothesis that CD133 expressing cells present within the bulk human endometrial tumor cell population have enhanced tumor initiating capacity, it is highly unlikely that all tumor initiating cells express CD133 or that all CD133^+ ^cells are tumorigenic. Regardless, it is well accepted that patients with advanced stage endometrial cancer have poorer prognosis due to tumors that may be refractory to chemotherapy and an increased incidence of recurrent disease. It remains to be determined whether an increasing level of CD133^+ ^tumor initiating cells, presumably in part due to hypomethylation of the CD133 promoter region contributes to the pathobiology of the advanced stage tumor or is an indirect consequence of disease progression.

## Competing interests

The authors declare that they have no competing interests.

## Authors' contributions

AMF, LZ, RF and BRR conceived the study and participated in its design. AMF, LZ and MDC conducted the study. VAT and SEB were responsible for consenting patients and initial tissue processing. PAS processed tissue and isolated nucleotides. DRB isolated nucleotides from paraffin blocks and edited the manuscript. GM conducted aCGH. LZ served as the pathologist in this study. AMF, RF and BRR were primarily responsible for writing the manuscript. All authors read and approved the final manuscript.
